# Retinol Binding Protein-4 Levels and Non-alcoholic Fatty Liver Disease: A community-based cross-sectional study

**DOI:** 10.1038/srep45100

**Published:** 2017-03-23

**Authors:** Xuechen Chen, Tianran Shen, Qing Li, Xu Chen, Yanping Li, Dan Li, Gengdong Chen, Wenhua Ling, Yu-ming Chen

**Affiliations:** 1 Department of Nutrition, School of Public Health, Sun Yat-Sen University, Guangzhou, P. R. China; 2 Guangdong Provincial Key Laboratory of Food, Nutrition and Health, Guangzhou, P. R. China; 3 Department of Epidemiology, School of Public Health, Guilin Medical University, Guilin, P. R. China

## Abstract

Previous reports on the association between retinol binding protein 4 (RBP4) and nonalcoholic fatty liver disease (NAFLD) were controversial. This study aimed to investigate the association between the serum RBP4 levels and occurrence of NAFLD in Chinese population. In total, 2938 participants aged 40–75 years were involved in this community-based cross-sectional study. General information, lifestyle factors, serum levels of RBP4 and the presence of NAFLD were determined. Patients with NAFLD had significantly higher concentrations of RBP4 (37.9 ± 6.8 μg/ml) than did non-NAFLD controls (35.0 ± 6.7 μg/ml) (*P* < 0.001). The odds ratios (ORs) of NAFLD for the highest (vs. lowest) quartile of RBP4 were 1.884 (95% CI: 1.391, 2.551) for females (*P* < 0.001), and 2.107 (95% CI: 1.357, 3.273) for male participants (*P* < 0.01) after adjusting for related factors. The serum RBP4 levels were positively associated with the prevalence of NAFLD in middle-aged and elderly Chinese people, and Homeostatic model assessment-insulin resistance (HOMA-IR), trunk fat, the waist-to-hip ratio (WHR), systolic blood pressure (SBP), fasting insulin, high density lipoprotein cholesterol (HDL-C) and triglycerides (TG) might be implicated in the pathogenesis of RBP4 in NAFLD.

Non-alcoholic fatty liver disease (NAFLD) is defined as a condition in which more than 5% of the hepatocytes exhibit macroscopic steatosis with no cause of secondary hepatic fat accumulation such as increased alcohol consumption, the use of steatogenic medications or hereditary disorders^1^. The estimated worldwide prevalence of NAFLD ranges approximately from 6% to 35%, with a median of 20% in the general population, based on various assessment methods^2^. NAFLD encompasses simple steatosis and non-alcoholic steatohepatitis (NASH), which can progress to fibrosis and, finally, cirrhosis, even with hepatic cellular cancer (HCC)^3^. Furthermore, in most patients, NAFLD is often associated with an increased risk of obesity, insulin resistance (IR), and hyperlipidemia, all of which are components of the metabolic syndrome^4^. NAFLD, with its high prevalence and severe complications, has, accordingly, become the most common liver disease in adults and an alarming public health problem globally.

The contribution of retinol binding protein 4 (RBP4) in obesity and IR was first discovered in adipose-*Glut4*^−/−^ mice^5^, after which the results of a human study showed that elevated serum RBP4 levels were correlated with the magnitude of IR and metabolic risk factors in lean, obese, and diabetic subjects^6^. The main function of RBP4, which is highly expressed in the liver, is to transport retinol from the liver stores to extra-pancreatic tissues^7^. Adipose tissue (AT) has the second highest rate of expression^8^. A few studies have demonstrated that elevated RBP4 is a risk factor associated with NAFLD^9^,^10^. Several human studies have shown that the serum RBP4 levels have a positive association with the degree of liver fat accumulation and liver enzyme levels, including serum alanine aminotransferase (ALT), aspartate aminotransferase (AST) and γ-glutamyltranspeptidase (GGT)^11^,^12^.

However, the data in the literature concerning the role of RBP4 in IR and NAFLD are inconsistent. The serum RBP4 levels were not associated with whole-body or hepatic IR in Mexican Americans^13^. In addition, several studies have shown that the serum RBP4 levels were not different between the steatosis group and controls^14^,^15^. Furthermore, serum RPB4 was found to be significantly lower in NAFLD patients than in controls^16^. Conflicting results from these studies mentioned above might be mainly because of the different populations as well as the limited samples of these studies. To date, the study with the largest sample to investigate the relationship between RBP4 and NAFLD was conducted in 748 school children, and only 219 of them volunteered to accept ultrasound examinations^17^. Therefore, a large-size human population is required to observe the association of serum RBP4 and the occurrence of NAFLD. Accordingly, we conducted a large community-based cross-sectional study to explore the association between RBP4 and NAFLD. Elucidating these questions can help find whether RBP4 predicts the development of NAFLD and aid in the identification of potential biomarkers to prevent NAFLD progression.

## Results

### Characteristics of participants

Our data showed that patients with NAFLD had higher levels of body mass index (BMI), waist-to-hip ratio (WHR), trunk fat percentage and serum ALT, fasting glucose, homeostatic model assessment-insulin resistance (HOMA-IR), triglycerides (TG) and uric acid (UA) as well as a high prevalence of high blood pressure (HBP) and diabetes mellitus (DM), but they had lower physical activities and serum high density lipoprotein cholesterol (HDL-C) than did those with non-NAFLD in both the female and male groups. NAFLD patients had higher low density lipoprotein cholesterol (LDL-C) than did non-NAFLD subjects only in the female group. There were no differences in household income and serum AST and total cholesterol (TC) between NAFLD and non-NAFLD participants divided by sex (Table 1).

### Association of serum RBP4 levels with metabolic risk factors

We next assessed the correlation between the serum RBP4 levels and a battery of known metabolic risk factors (Table 2). Our analysis revealed a significantly positive association between serum RBP4 and age, BMI, WHR, systolic blood pressure (SBP), diastolic blood pressure (DBP), trunk fat and several biochemical parameters, including fasting glucose, fasting insulin, and serum TG, ALT and UA in all participants (*P* < 0.001 for all above parameters). In addition, a negative association between RBP4 and the HDL-C levels was observed (*P* < 0.001). However, there was no significant association between RBP4 and SBP and fasting glucose in the male group among the variables mentioned above. A positive association between RBP4 and LDL-C was found only in female participants.

### Relationship between serum RBP4 and IR and other correlated metabolic risk factors

To investigate the relationship between serum RBP4 and IR as well as other correlated metabolic risk factors, multiple linear regression was performed using RBP4 as a dependent variable. As presented in Table 3, HOMA-IR, trunk fat, WHR, SBP, fasting insulin, HDL-C and TG were all selected as independent variables in all subjects before and after adjusting for age, sex, BMI (except for BMI, trunk fat and WHR), current smoking and drinking, physical activities, history of diabetes and hypertension, LDL-C, Lg (ALT), and UA (*P* < 0.001 for all of the above parameters). However, DBP and fasting glucose were just significant variables associated with the serum RBP4 levels before adjustment.

### Serum RBP4 levels in non-NAFLD subjects and NAFLD patients

**Figure 1:**
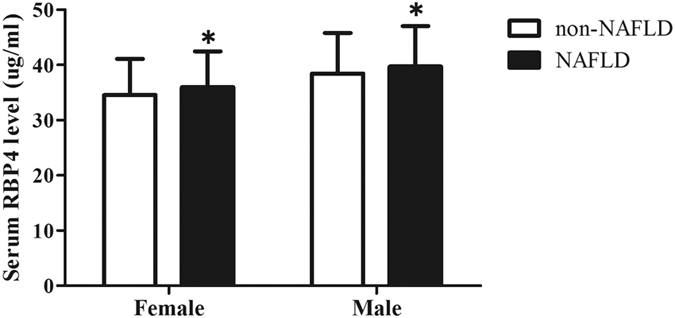
Serum RBP4 concentrations (μg/ml) in non-NAFLD subjects and NAFLD patients. *NAFLD vs. non-NAFLD subjects in both female and male groups adjusted by variables (included in model 3) in the general linear model, *P* < 0.001.

The serum RBP4 levels ranged from 13.3 to 67.9 μg/ml among the participants. The results demonstrated that patients with NAFLD had significantly higher RBP4 concentrations (37.9 ± 6.8 μg/ml) than did those with non-NAFLD (35.0 ± 6.7 μg/ml) (*P* < 0.001). After the adjustment for variables (included in model 3 of Table 4), the serum RBP4 levels in patients with NAFLD (36.0 ± 6.5 μg/ml) were significantly higher than those in control subjects (34.6 ± 6.5 μg/ml) of females and in males (39.7 ± 7.3 μg/ml vs. 38.4 ± 7.4 μg/ml) (both *P* < 0.001) (Fig. 1).

### Association of serum RBP4 with NAFLD

To explore the ability of RBP4 to predict the presence of NAFLD, multivariate logistic regression analysis was performed. We found that serum RBP4 levels were positively associated with NAFLD in all subjects [odds ratio (OR) for the highest quartile vs. lowest quartile, 3.481 (95% CI: 2.806–4.320, *P* < 0.001). As shown in Table 4, after adjusting for age, postmenopausal (females) and household income in both the female and male groups, the serum RBP4 levels were significantly positively associated with the prevalence of NAFLD (all *P* < 0.01). Furthermore, these associations remained robust after adjusting for the physical and metabolic parameters in models 2 and 3. The ORs of NAFLD for the highest (vs. lowest) quartile in model 3 were 1.884 (95% CI: 1.391, 2.551) for females (*P* < 0.001) and 2.107 (95% CI: 1.357, 3.273) for males (*P* < 0.01). The ROC analysis of serum RBP4 levels for the prediction of NAFLD can be found in Supplementary Fig. S1.

## Discussion

In this study, we confirmed a positive correlation between the serum RBP4 levels and the prevalence of NAFLD in a large, community-based, middle-aged and elderly Chinese population. It appears that the correlation is independent of IR, trunk fat and other metabolic factors, with no gender difference. Furthermore, our study demonstrated a significant association between the serum RBP4 levels and metabolic risk factors, including IR, dyslipidemia and obesity, in this cohort.

The current study revealed a surprisingly high prevalence of NAFLD, with 50% of the cases presenting ultrasonographical evidence of steatosis. The major reasons for the high prevalence of NAFLD are as follows: First, there was an increasing relative risk of NAFLD with older age groups that was independent of other factors^18^, and the protective effect of female hormones became lost during the postmenopausal period^19^. Second, although the ultrasonography used in this study was an established tool for NAFLD screening, inter- and intra-observer variability could not be ignored compared with liver biopsy^20^. Third, the substantially increased prevalence of fatty liver observed over the last decade was in parallel with regional trends in over-nutrition, diabetes, dyslipidemia and, especially, obesity^21^. Therefore, the increased prevalence of NAFLD might be due to the high proportion of overweight (73.9%) and obese (7.5%) subjects in our study.

The serum RBP4 levels were positively associated with the occurrence of NAFLD in the current study, consistent with the results of previous studies^9^,^10^,^12^. We noticed that the concentration of serum RBP4 varied by gender and was generally lower in women than in men. However, only the highest quartiles of RBP4 in the male group were significantly associated with a high risk of NAFLD, which indicated that there must be a protective element to neutralize the adverse effect of RBP4 in male patients. Several studies have hypothesized that the sex hormone testosterone might play a role in this situation^22^,^23^. The mechanisms underlying the association between testosterone and NAFLD are still largely unclear. One possible explanation is that testosterone is an insulin sensitizer and is thus a metabolic hormone^24^. In addition, testosterone may act, in part, via an effect on the key regulatory lipogenic enzymes to protect against hepatic steatosis^25^.

However, there are some controversies among the previously published studies on the relationship between RBP4 and NAFLD^14^,^16^. The reasons why we have inconsistent findings can be roughly summarized as follows: (1) Circulating RBP4 is cleared by the kidney glomerular filtration, and kidney dysfunction probably contributes to high levels of serum RBP4^26^. Furthermore, the risk of kidney disease is higher in patients with metabolic syndrome^27^, which, if it is not strictly controlled in the subjects, will confound the relationship between NAFLD and RBP4. (2) The existence of substantial differences in the characteristics of study populations, as well as different methodologies of RBP4 examination, may lead to inconsistent results^28^. (3) RBP4 was negatively associated with ALT in all participants and was especially higher in patients with NAFLD^9^,^10^,^12^. Elevated serum ALT is usually used as a cytolytic marker of the liver because it will be released into the serum when the liver is injured^29^. In parallel, RBP4 may also increase in serum when hepatocytes damage occurs. It is noteworthy that we initially excluded patients with CKD to reduce the bias caused by renal dysfunction. Additionally, our analysis included a nearly 3000-member community-based Chinese population that could reduce the measurement bias and balance the confounding factors. However, considering the feasibility and invasiveness of liver biopsy, we chose abdominal ultrasonography, which could not eliminate the effect of liver damage.

RBP4 is synthesized primarily in the liver, with its secretion being dependent on the retinol concentrations. RBP4 forms a 1:1:1 complex with retinol (holo-RBP4) and transthyretin in circulation. Its most well-defined function is to transport retinol to peripheral tissues in circulation^7^. After releasing retinol into the target cells, the remaining apo-RBP4 is rapidly filtered through the glomeruli and reabsorbed in the proximal tubular cells and catabolized^30^. Studies on whether RBP4 is a cause or just relevant to NAFLD have not achieved consistent results. The role of RBP4 in IR and lipid mechanism may explain the interaction between RBP4 and NAFLD. First, RBP4 may influence the insulin signaling pathway through the inhibition of IRS1 (insulin receptor substrate 1) phosphorylation in primary human adipocytes and skeletal muscle in mice^5^,^31^. Second, RBP4 could cause IR by stimulating the production of proinflammatory cytokines, which finally leads to intracellular fat accumulation^32^,^33^. Third, the impact of RBP4 on stimulating lipogenesis in hepatocytes has been investigated. It might initially up-regulate peroxisome proliferator activated receptor-γ coactivator 1-β (PGC1β) and finally promote the transcription of downstream target lipogenic genes, thereby stimulating *de novo* lipogenesis *in vitro* and *in vivo*^34^.

Some limitations merit consideration. The cross-sectional observational design was one of the major limitations in our investigation, which precluded causal inferences. In addition, we might still ignore many other mediators in different biological pathways involved in the pathogenesis of NAFLD. For example, it is well recognized that a nonsynonymous single nucleotide polymorphism rs738409 (I148M) in patatin-like phospholipase domain-containing protein 3 (*PNPLA3*) predisposes a patient to susceptibility to chronic liver disease^35^,^36^,^37^. PNPLA3 promotes the extracellular release of retinol from lipid droplets in hepatic stellate cells, and I148M mutation results in a loss of this function^38^. Moreover, the circulating concentrations of RBP4 were declined in overweight/obese individuals with NAFLD carrying the PNPLA3 148 M mutant^38^, which could be a confounding factor in the current study. Abdominal ultrasonography was used as a noninvasive modality to screen the general population to detect NAFLD because liver biopsy is invasive, difficult to perform in large populations and carries some risk of complications^39^. Therefore, the absence of histological confirmation of the liver status could be another major weakness of this study. Except for these factors, the method used to measure the RBP4 concentration (enzyme immunoassay) could underestimate its level^28^. Finally, most of our participants were middle-aged and elderly people in a Chinese population, which limited the generality of the results.

In conclusion, our study provides clinical evidence revealing that the serum concentrations of RBP4 were elevated in NAFLD patients in a Chinese population. These findings indicated that RBP4 might be a noninvasive molecular biomarker that detects the presence of NAFLD in middle-aged and elderly population. Additional large-scale studies are needed to explore these associations in other populations. However, further work is needed to determine the physiological role of RBP4 in the liver, which is crucial for clarifying the causal relationship between RBP4 and NAFLD and seeking efficacious therapies to improve NAFLD.

## Methods

### Study population

Our study was performed on a community-based, prospective cohort, namely the Guangzhou Nutrition and Health Study (GNHS). This cohort was established to investigate the potential association between nutrition, diet, and genetic factors, along with their interactions, with non-communicable chronic diseases. Between June 2008 and June 2010, 3169 participants aged 40–75 years who had at least a 5-year continuous residence in Guangzhou were initially recruited into this study. Of these participants, 2465 were followed up between April 2011 and March 2013. An additional 871 participants were recruited between March 2013 and August 2013.

**Figure 2:**
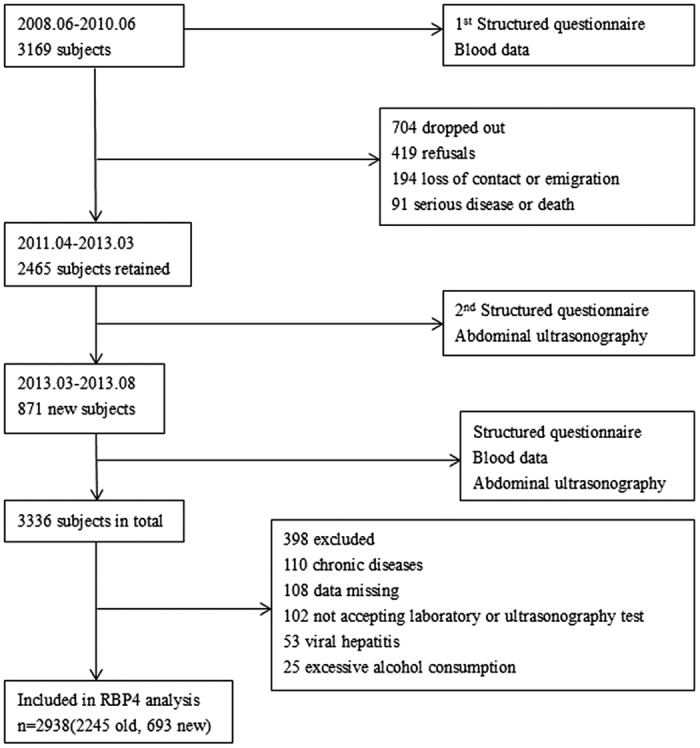
Flow-chart of the recruited participants.

All of the participants underwent a comprehensive physical examination, routine biochemical analysis of blood, hepatitis virus test and B-scan ultrasonography. Subjects with the following conditions were excluded from the study: excessive alcohol consumption (≥140 g/wk for males or ≥70 g/wk for females^40^); viral hepatitis [with positive hepatitis B surface antigen (HbsAg) or positive anti-HCV antibody^40^]; biliary obstructive diseases; drug- or toxin-induced liver diseases; autoimmune hepatitis; chronic inflammatory disease; the presence of severe medical diseases such as cancer, stroke and heart failure; current treatment with systemic corticosteroids, anti-inflammatory and lipid-lowering therapy; and pregnancy. In total, 2938 participants, followed up from the original cohort (n = 2245) and newly recruited participants (n = 693), were finally analyzed in this study (the study flow-chart is presented in Fig. 2).

The study protocol was approved by the Ethics Committee of the School of Public Health at Sun Yat-sen University. Written informed consent was obtained from all participants at the initial enrollment and at each of the follow-ups. Therefore, the study was performed in accordance with the ethical standards laid down in the 1964 Declaration of Helsinki and its later amendments.

### Data collection

A structured questionnaire was designed to collect the participants’ socio-demographic characteristics (e.g., age, sex and household income), health-related lifestyle factors (e.g., smoking, alcohol drinking and physical activity) and history of chronic disease by trained staff through face-to-face interviews. Participants wore lightweight clothes and no shoes for the measurement of weight and height. BMI was calculated as the weight (kg)/height^2^ (m^2^) (BMI ≥ 24 for overweight and ≥28 for obesity according to Chinese classification^41^). Daily physical activity was estimated using a 24-h physical activity questionnaire, and the metabolic equivalent (MET) intensity was also calculated^42^. The fat mass (FM) and %FM of the trunk region were quantified by Dual-energy X-ray absorptiometry (Discovery W; Hologic Inc., Waltham, MA, USA). The trunk region was defined as the area between an upper horizontal border below the chin, and a lower border was formed by oblique lines passing through the hip joints.

The data on current alcohol consumption was obtained by a self-administered questionnaire. Individuals were first questioned for whether they consume alcohol currently. Participants who answered ‘no’ were considered non-drinkers. Participants who responded ‘yes’ were further asked about the average drinking frequency per week and the average volume of wine or beer consumed per drinking day. The average amount of alcohol (g/week) = the average drinking frequency* average amount of alcohol consumed per drinking day [%ABV (alcohol by volume)*0.79336 (g/ml)*Volume (ml)].

### Abdominal ultrasonography

All ultrasound examinations, using a Doppler sonography machine (Sonoscape SSI-5500, Shenzhen, China) with a 3.5-MHz probe, were performed to diagnose NAFLD by a single experienced radiologist who was blinded to the laboratory and clinical data. The diagnosis of NAFLD was based on standard criteria issued by the Fatty Liver Disease Study Group of the Chinese Liver Disease Association^43^.

### Biochemical measurements

Venous blood samples were collected from the participants after overnight fasting. The serum was separated into several aliquots and stored at −80 °C within 2 hours. Colorimetric methods were used to measure fasting glucose, TG, TC, HDL-C, LDL-C, AST, ALT and UA in a Hitachi 7600-010 automated analyzer (Hitachi, Tokyo, Japan). IR was evaluated using the homeostasis model assessment (HOMA) in which HOMA = fasting glucose (mmol/L) × fasting insulin (mU/L)/22.5. The diagnosis of virus infection is established through serological testing using chemiluminescence immunoassay. All patients with positive HbsAg or positive anti-HCV antibody were also under the detection of HBV DNA and HCV RNA in serum using polymerase chain reaction (PCR).

Serum RBP4 was measured using enzyme-linked immunosorbent assay (ELISA) kit (Adipogen, San Diego, California, USA), and the absorbance was determined using a microplate spectrophotometer (BIO-TEK, Winooski, Vermont, USA). The lowest level of RBP4 that can be detected by this assay is 380 pg/ml, and the intra-assay coefficients of variation for RBP4 were 3.59%.

### Statistical analysis

Descriptive statistics were computed for all variables. The normality test of Kolmogorov-Smirnov was performed to assess whether the data were normally distributed. The means ± SDs or medians [25th, 75th percentiles] were used to describe continuous factors. For categorical variables, the frequencies and percentages were estimated. Categorical data were compared using Chi-squared test. Student’s *t*-test was used for parametric data comparison, and the Mann–Whitney *U*-test was used for non-parametric data.

The association between the serum RBP4 levels and metabolic risk factors was estimated using Pearson’s or Spearman’s correlation coefficient. Logistic regression analyses were used to estimate the ORs and 95% confidence intervals (CIs) in three models for the risk of NAFLD with increasing quartiles of serum RBP4 levels, using the lowest quartile as the reference group. Analysis of covariance (ANCOVA) assessed the serum RBP4 levels of NAFLD patients compared with non-NAFLD subjects stratified by sex. A two-tailed *P*-value < 0.05 was considered statistically significant. All statistical procedures were performed using SPSS Statistics (version 22.0, SPSS Inc, Chicago, IL).

## Additional Information

**How to cite this article:** Chen, X. *et al*. Retinol Binding Protein-4 Levels and Non-alcoholic Fatty Liver Disease: A community-based cross-sectional study. *Sci. Rep.* **7**, 45100; doi: 10.1038/srep45100 (2017).

**Publisher's note:** Springer Nature remains neutral with regard to jurisdictional claims in published maps and institutional affiliations.

## Supplementary Material

Supplementary Information

## Figures and Tables

**Table 1:** Demographic characteristics, physical and metabolic measurements by sex and NAFLD status.

	Female			Male		
	non-NAFLD	NAFLD	*P*	non-NAFLD	NAFLD	*P*
N	1022	997		436	483	
Age (years)	59.5 ± 5.6	60.2 ± 5.3	0.003	63.0 ± 6.5	62.1 ± 6.0	0.033
Postmenopausal	1010 (98.8%)	973 (97.6%)	0.036	—	—	—
Household income, yuan/month/person			0.703			0.357
<4000	879 (87.1%)	852 (87.1%)		346 (79.5%)	363 (75.6%)	
4000–6000	84 (8.3%)	75 (7.7%)		61 (14.0%)	82 (17.1%)	
>6000	46 (4.6%)	51 (5.2%)		28 (6.4%)	35 (7.3%)	
Current smoking	3 (0.3%)	5 (0.5%)	0.502	65 (14.9%)	108 (22.4%)	0.004
Current drinking	5 (0.5%)	5 (0.5%)	1	153 (35.1%)	173 (35.8%)	0.836
Physical activities, MET/d	34.4 ± 5.5	33.8 ± 5.2	0.007	34.4 ± 6.1	33.3 ± 6.1	0.007
BMI (kg/m^2^)	22.0 ± 2.6	24.8 ± 3.1	<0.001	22.4 ± 2.5	25.3 ± 2.9	<0.001
WHR	0.90 ± 0.07	0.93 ± 0.07	<0.001	0.91 ± 0.06	0.95 ± 0.05	<0.001
Trunk fat percentage (%)	34.3 ± 5.5	38.5 ± 4.1	<0.001	24.8 ± 5.4	29.9 ± 4.3	<0.001
HBP	201 (19.7%)	358 (35.9%)	<0.001	124 (28.4%)	174 (36.0%)	0.006
DM	58 (5.7%)	86 (8.6%)	0.008	35 (8.0%)	54 (11.2%)	0.205
ALT (U/L)	16 [11,18]	19 [12,22]	<0.001	15 [12,20]	19 [14,25]	<0.001
AST (U/L)	20 [16,22]	20 [15,22]	0.195	19 [16,22]	19 [16,22]	0.648
Fasting glucose (mmol/L)	4.85 [4.31,5.20]	5.11 [4.40,5.39]	<0.001	4.82 [4.40,5.38]	4.92 [4.50,5.49]	0.014
HOMA-IR	1.55 [0.94,1.89]	2.65 [1.50,3.24]	<0.001	1.16 [0.85,1.76]	2.11 [1.45,3.05]	<0.001
TG (mmol/L)	1.28 [0.81,1.47]	1.73 [1.05,2.06]	<0.001	1.15 [0.80,1.55]	1.44 [1.04,2.10]	<0.001
TC (mmol/L)	5.73 ± 1.05	5.70 ± 1.03	0.42	5.37 ± 1.03	5.24 ± 0.98	0.065
HDL-C (mmol/L)	1.63 ± 0.42	1.38 ± 0.35	<0.001	1.38 ± 0.36	1.18 ± 0.31	<0.001
LDL-C (mmol/L)	3.62 ± 0.91	3.71 ± 0.88	0.029	3.42 ± 0.87	3.40 ± 0.92	0.763
UA (μmol/L)	314.9 ± 70.9	346.4 ± 77.3	<0.001	387.4 ± 87.3	403.5 ± 86.8	0.005

**Table 2:** Correlation between RBP4 and metabolic risk factors.

Variables	Total (n = 2938)		Female (n = 2019)		Male (n = 919)	
	*r*	*P*	*r*	*P*	*r*	*P*
Age (years)	0.065	<0.001	0.055	0.014	−0.067	0.043
BMI (kg/m^2^)	0.186	<0.001	0.189	<0.001	0.141	<0.001
WHR	0.183	<0.001	0.167	<0.001	0.142	<0.001
SBP (mmHg)	0.123	<0.001	0.110	<0.001	0.053	0.109
DBP (mmHg)	0.121	<0.001	0.090	<0.001	0.068	0.040
Fasting glucose (mmol/l)	0.104	<0.001	0.130	<0.001	0.056	0.091
Fasting insulin (μU/ml)	0.211	<0.001	0.268	<0.001	0.171	<0.001
HOMA-IR	0.213	<0.001	0.268	<0.001	0.160	<0.001
TG (mmol/l)	0.357	<0.001	0.368	<0.001	0.352	<0.001
TC (mmol/l)	−0.008	0.672	0.036	0.105	0.054	0.104
LDL-C (mmol/l)	−0.002	0.923	0.047	0.035	0.008	0.809
HDL-C (mmol/l)	−0.270	<0.001	0.248	<0.001	−0.143	<0.001
ALT (U/L)	0.161	<0.001	0.128	<0.001	0.160	<0.001
AST (U/L)	0.008	0.646	−0.014	0.534	0.054	0.103
Trunk Fat (kg)	0.131	<0.001	0.212	<0.001	0.129	<0.001
UA (μmol/L)	0.312	<0.001	0.268	<0.001	0.200	<0.001

**Table 3:** Association between serum RBP4 and IR as well as other correlated metabolic risk factors, using multiple linear regression analysis.

	Unadjusted		Adjusted	
	*β* ± SE	*P*	*β* ± SE	*P*
HOMA-IR	4.74 ± 0.46	<0.001	2.86 ± 0.54	<0.001
Trunk fat (kg)	0.31 ± 0.044	<0.001	0.29 ± 0.044	<0.001
WHR	19.15 ± 1.90	<0.001	7.55 ± 2.08	<0.001
SBP (mmHg)	0.046 ± 0.007	<0.001	0.018 ± 0.007	0.009
DBP (mmHg)	0.081 ± 0.012	<0.001	0.018 ± 0.012	0.152
Fasting glucose (mmol/L)	6.81 ± 1.55	<0.001	1.49 ± 1.50	0.323
Fasting Insulin (μU/ml)	5.18 ± 0.52	<0.001	3.28 ± 0.60	<0.001
HDL-C (mmol/L)	−4.58 ± 0.30	<0.001	−2.77 ± 0.32	<0.001
TG (mmol/L)	10.42 ± 0.52	<0.001	8.69 ± 0.53	<0.001

Fasting glucose, fasting insulin, HOMA-IR and TG were put into linear regression after log transformation. All variables were adjusted for age, sex, BMI (except for BMI, trunk fat and WHR), Current smoking and drinking, Physical activities, history of diabetes and hypertension, LDL-C, lg (ALT), UA.

**Table 4:** Adjusted odds ratios (95% confidence interval) of NAFLD according to the sex and quartiles of serum RBP4 in total participants using Adjusted Logistic Regression.

	RBP4 (μg/ml)				*P* for trend
	Quartile 1	Quartile 2	Quartile 3	Quartile 4	
**Female**	<31.05	31.06–35.19	35.20–39.35	>39.35	
Model 1	1	1.464 (1.130–1.897)	2.852 (2.200–3.697)	3.646 (2.801–4.746)	<0.001
Model 2	1	1.399 (1.073–1.825)	2.579 (1.978–3.363)	3.188 (2.434–4.175)	<0.001
Model 3	1	1.140 (0.855–1.520)	1.930 (1.444–2.580)	1.884 (1.391–2.551)	<0.001
**Male**	<34.37	34.38–38.91	38.92–43.43	>43.43	
Model 1	1	1.650 (1.135–2.399)	1.674 (1.151–2.435)	3.119 (2.113–4.602)	<0.001
Model 2	1	1.370 (0.919–2.043)	1.354 (0.910–2.015)	2.500 (1.654–3.779)	<0.001
Model 3	1	1.294 (0.856–1.955)	1.240 (0.817–1.881)	2.107 (1.357–3.273)	0.008

Model 1: Adjusted for age, postmenopausal (for female) and household income.

Model 2: Adjusted for variables in Model 1 plus waist-to-hip ratio (WHR), trunk fat, current smoking and drinking, physical activity, hypertension and diabetes.

Model 3: Adjusted for variables in Model 2 plus fasting glucose, HOMA-IR, TG, HDL-C, ALT, and UA.
