# Supplementary material for: Retinol Binding Protein-4 Levels and Non-alcoholic Fatty Liver Disease: A community-based cross-sectional study
Source: Sci Rep. 2017 Mar 23;7:45100. doi: 10.1038/srep45100 (PMC5362806; doi:10.1038/srep45100)
Supplement: Supplementary Information [file srep45100-s1.pdf]

Supplemental materials

Retinol Binding Protein-4 Levels and Non-alcoholic Fatty Liver Disease: A community-based cross-sectional study

Short title: RBP4 and NAFLD

Xuechen Chen[1,2#], Tianran Shen[1,2#],Qing Li[3], Xu Chen[1,2], Yanping Li[1,2],Dan Li[1,2], Gengdong Chen[2], Wenhua Ling[1,2]*, Yuming Chen[1,2]*

[1] Department of Nutrition, School of Public Health, Sun Yat-Sen University, Guangzhou, P. R. China

[2] Guangdong Provincial Key Laboratory of Food, Nutrition and Health, Guangzhou, P. R. China

[3]Department of Epidemiology, School of Public Health, Guilin Medical University, Guilin, P. R. China

[#] These authors contributed equally to this work.

*Correspondent authors

Dr. Wenhua Ling, Department of Nutrition, School of Public Health, Sun Yat-Sen University, Guangzhou, 510080, P. R. China

Tel.: 86-20-87331597

Fax: 86-20-87330446

E-mail: lingwh@mail.sysu.edu.cn

Dr. Yuming Chen, School of Public Health, Sun Yat-Sen University, Guangzhou, 510080, P. R. China

Tel.: 86-20-87330605

Fax: 86-20-87330605

E-mail: chenyum@mail.sysu.edu.cn

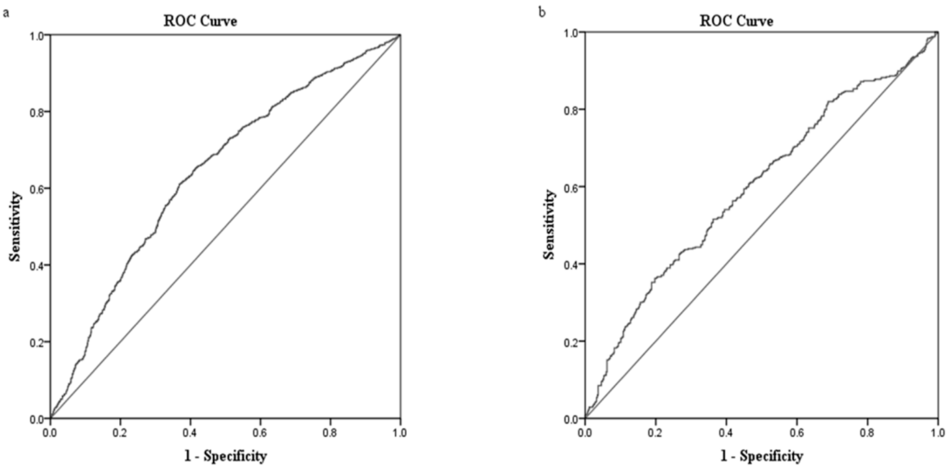

Supplemental Figure S1 ROC analysis of serum RBP4 levels for the prediction of NAFLD

a) ROC analysis for female individuals. The area under the ROC curve was 0.641 ±0.012 (95% CI: 0.617–0.665, *p*<0.001).The cutoff value of serum RBP4 was 35.34 μg/ml. The sensitivity was 60.9%, and the specificity was 63.1%.

b) ROC analysis for male individuals. The area under the ROC curve was 0.598 ±0.019 (95% CI: 0.561–0.634, *p*<0.001) .The cutoff value of serum RBP4 was 42.86 μg/ml. The sensitivity was 36.2%, and the specificity was 80.3%.
